# Crystal structure of (*E*)-1,2-diferrocenyl-1,2-bis(furan-2-yl)ethene

**DOI:** 10.1107/S2056989018005078

**Published:** 2018-04-06

**Authors:** Anthony Linden, Róża Hamera-Fałdyga, Grzegorz Mlostoń, Heinz Heimgartner

**Affiliations:** a Department of Chemistry, University of Zurich, Winterthurerstrasse 190, CH-8057 Zurich, Switzerland; b Department of Organic and Applied Chemistry, University of Łódź, Tamka 12, PL-91-403 Łódź, Poland

**Keywords:** crystal structure, ethenes, ferrocene

## Abstract

The title compound, [Fe_2_(C_5_H_5_)_2_(C_20_H_14_O_2_)], is the product of a new synthetic route towards tetra­aryl/hetaryl-substituted ethenes that reduces the occurrence of side-products. In the crystal, the mol­ecule is centrosymmetric and the cyclo­penta­dienyl (Cp) rings are nearly coplanar and aligned slightly closer to a staggered conformation than to an eclipsed one. The ethene plane is tilted by 32.40 (18)° with respect to that of the substituted Cp ring and by 63.19 (19)° with respect to that of the furan ring. C—H⋯π inter­actions link the mol­ecules into a three-dimensional supramolecular framework.

## Abstract 2

The title compound is the product of a new synthetic route towards tetra­aryl/hetaryl-substituted ethenes that reduces the occurrence of side-products. In the crystal, the mol­ecule is centrosymmetric and the cyclo­penta­dienyl rings are nearly coplanar and aligned slightly closer to a staggered conformation than to an eclipsed one.

## Chemical context   

Tetra­substituted ethenes bearing aryl, hetaryl or ferrocenyl groups are of current inter­est, as many of them find applications as novel materials for photooptics, electronics, crystal engineering and as new medications (Astruc, 2017 ▸). Ethene derivatives with a ferrocenyl unit on one or both C atoms of the alkene deserve special attention. Prominent representatives of the first type are ferrocifene {1-[4-(2-di­methyl­amino­eth­oxy)phen­yl]-1-phenyl-2-ferrocenylbut-1-ene} and its di-OH analogue, which are known as potent, organometallic anti­tumor drugs (Jaouen *et al.*, 2015 ▸; Resnier *et al.*, 2017 ▸). On the other hand, dimethyl (*Z*)-2,3-diferrocenylbut-2-enedioate displays inter­esting redox and solvatochromic properties (Solntsev *et al.*, 2011 ▸). As typical procedures for the preparation of tetra­substituted ethenes containing a ferrocenyl substituent, conversions of the corresponding ketones under the McMurry reaction conditions (Top *et al.*, 1997 ▸) or reductive coupling using low-valent titanium agents are recommended (Dang *et al.*, 1990 ▸). In both cases, the reported yields are satisfactory to good, but a serious disadvantage is the formation of side-products. Recently, we reported a new approach to tetra­aryl/hetaryl-substituted ethenes *via* desilyl­ation of 2-(tri­methyl­sil­yl)-4,4,5,5-tetra­aryl/hetaryl-1,3-di­thiol­anes, obtained from diar­yl/hetaryl ­thio­ketones by treatment with (tri­methyl­sil­yl)diazo­methane (TMS-CHN_2_) at low temperature (Mlostoń *et al.*, 2017 ▸). The mechanism of this unusual conversion was explained by the assumption that the *in situ*-generated 1,3-di­thiol­ane anion undergoes a spontaneous cyclo­elimination ([3 + 2]-cyclo­reversion) to give the di­thio­formate anion and the corresponding tetra­substituted ethene derivative. The same method was applied for the preparation of some ferrocen­yl/hetaryl-substituted eth­enes (Mlostoń *et al.*, 2018 ▸).

Here we report the analogous synthesis and crystal structure of the known title compound, (*E*)-**1** [Chem scheme1], with m.p. 485–487 K. For the previously described synthesis of this product (Dang *et al.*, 1990 ▸), a m.p. of 489–491 K and a yield of 17% were reported and the authors tentatively assigned the (*E*)-configuration to the obtained compound. In our case, single crystals of (*E*)-**1** were grown from hexa­ne/CH_2_Cl_2_ and used for an X-ray diffraction analysis, from which the previous tentatively postulated structure of the obtained isomer could be confirmed.
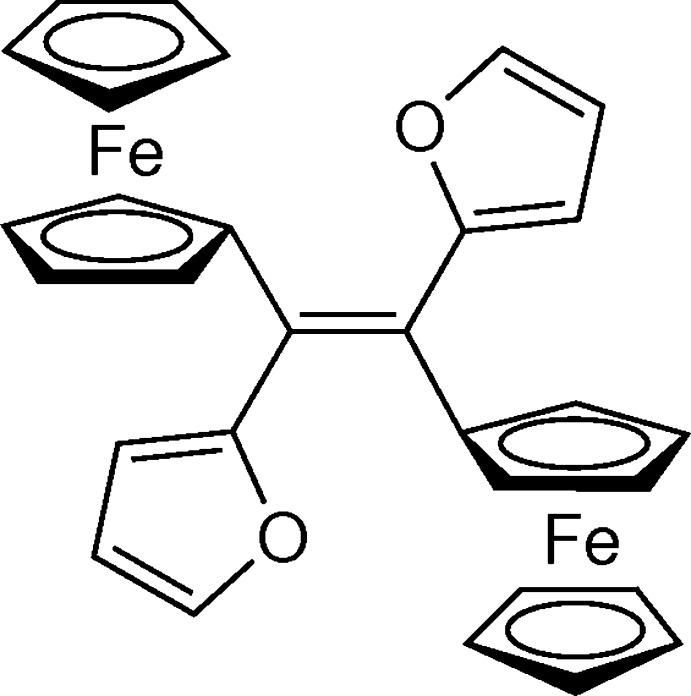


## Structural commentary   

**Figure 1:**
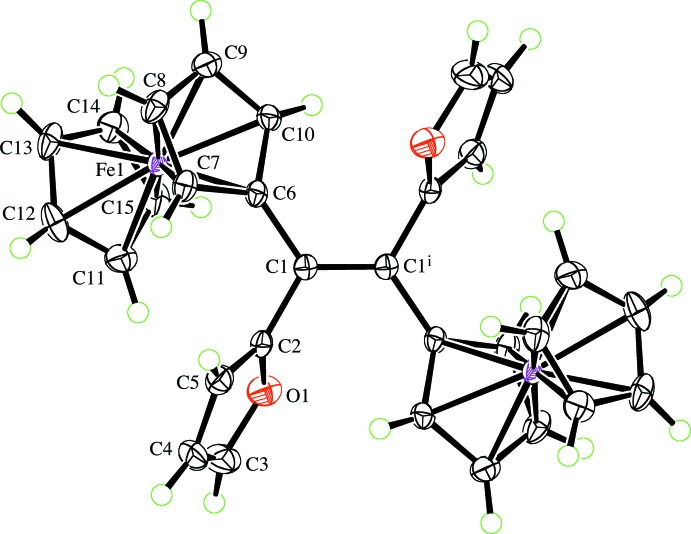
The mol­ecular structure of the title compound, (*E*)-**1**, showing the atom-numbering scheme. Displacement ellipsoids are drawn at the 50% probability level. Symmetry code: (i) −*x* + 1, −*y* + 1, −*z* + 1.

The mol­ecule of (*E*)-**1** [Chem scheme1] sits across a crystallographic centre of inversion and is shown in Fig. 1 ▸. Within the asymmetric unit, the Fe atom sits very well centred between the cyclo­penta­dienyl (Cp) rings with all Fe—C distances in the range 2.0352 (17)–2.0712 (16) Å. The Cp C—C bond lengths [mean 1.435 (2) Å] involving the substituted C atom, C6, are very slightly elongated compared with the other C—C distances [mean 1.418 (3) Å]. Other bond lengths and angles are unremarkable. The two Cp rings are aligned slightly closer to a staggered conformation than to an eclipsed one, with the ring rotation from perfectly eclipsed being 20.6 (2)° (18° is the half-way point between eclipsed and staggered). The dihedral angle between the planes of the two Cp rings in the ferrocenyl entity is only 4.08 (11)° and ethene atom C1 is coplanar with the Cp ring to which it is bonded. However, the ferrocenyl entity is tilted with respect to the ethene plane, with a dihedral angle between the plane of the substituted Cp ring and that of the ethene plane of 32.40 (18)°. The dihedral angle between the substituted Cp ring and the adjacent furan ring is 53.46 (11)°, while that between the plane of the furan ring and the ethene plane is 63.19 (19)°. The planes of the two furan rings are necessarily parallel because of the centre of inversion.

## Supra­molecular features   

**Figure 2:**
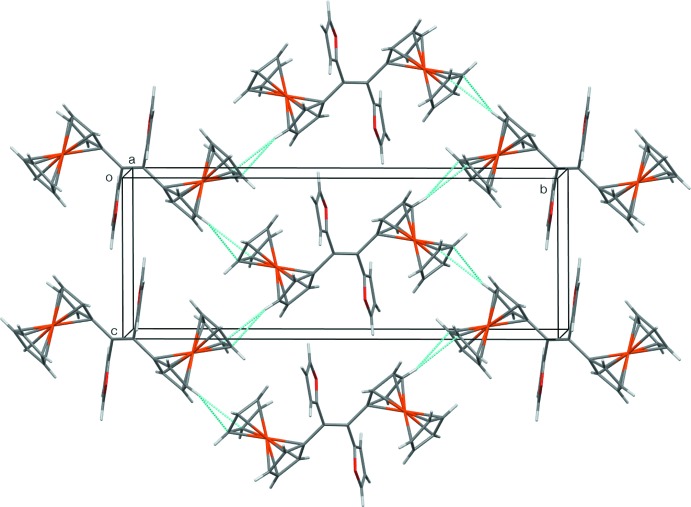
The sheets of mol­ecules lying parallel to the (101) plane formed by the C8—H⋯π inter­actions.

**Figure 3:**
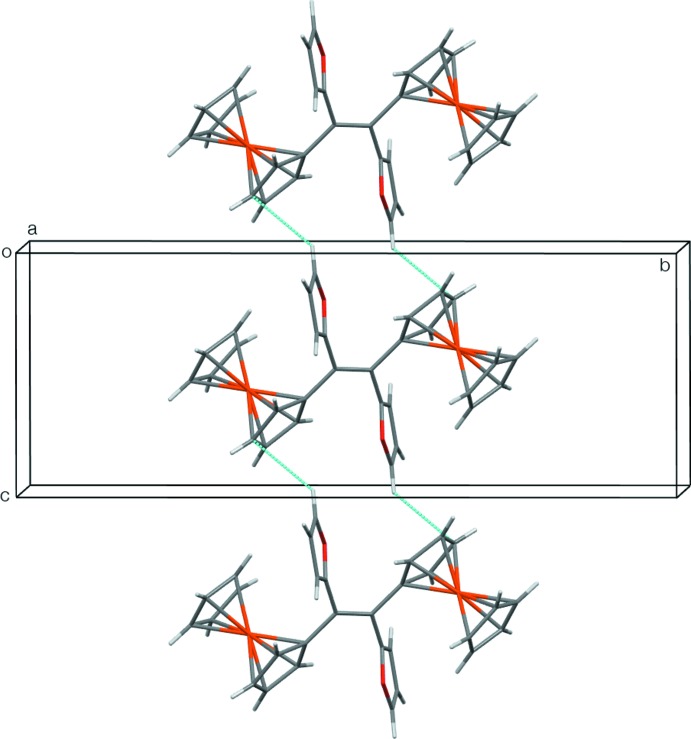
The ladder motif running parallel to [001] formed by the C3—H⋯π inter­actions.

There are no significant C—H⋯O or π–π inter­actions, but some weak C—H⋯π inter­actions are present (Table 1 ▸). C8—H of the substituted Cp ring has an edge-on intermolecular inter­action with the unsubstituted Cp ring at *x* + 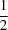, −*y* + 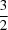, *z* − 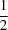. The extension of this inter­action through the mol­ecular centre of inversion leads to sheets of mol­ecules, which lie parallel to the (101) plane (Fig. 2 ▸). The furan ring, *via* C3—H, has an edge-on intermolecular C—H⋯π inter­action with the substituted Cp ring at *x*, *y*, *z* + 1. This inter­action leads to double-stranded chains or ladders, in which the mol­ecule acts as the ladder rungs; the chains run parallel to the [001] direction (Fig. 3 ▸). Finally, C10—H of the substituted Cp ring inter­acts intra­molecularly with the π-system of the furan ring at −*x* + 1, −*y* + 1, −*z* + 1 on the opposite side of the mol­ecule. This latter inter­action is quite short, but has a sharp angle at the H atom (Table 1 ▸), so the arrangement might just be a consequence of the mol­ecular conformation. The mol­ecular inversion symmetry, in combination with the two types of inter­molecular inter­actions, links the mol­ecules into a three-dimensional supramolecular framework.

## Database survey   

The Cambridge Structural Database (CSD, Version 5.39 with February 2018 updates; Groom *et al.*, 2016 ▸) contains one entry for a 1,1-diferrocenylethene [1,1-bis­(1′′,2′′,3′′,4′′,5′′-penta­methyl­ferrocen-1′-yl)ethene, CSD refcode CIJQAN, Heigl *et al.*, 1999 ▸] and 24 entries involving related 1,2-diferrocenyl­ethenes, 10 of which are (*E*)-isomers. The archetypal structure is (*E*)-1,2-diferrocenylethene (REBDAD, Denifl *et al.*, 1996 ▸), in which the Cp rings of the ferrocenyl entities adopt an almost perfectly eclipsed arrangement. With the exception of 1-(1′-benzoyl­ferrocen­yl)-2-ferrocenylethene and 1-(1′-(4-meth­oxy­benzo­yl)ferrocen­yl)-2-ferrocenylethene (OJUWUN and OJUXAU, Roemer *et al.*, 2016 ▸), in which the ferrocenyl Cp rings lie close to a staggered arrangement, all of the other structures of mol­ecules with the (*E*)-configuration display Cp arrangements that are much closer to eclipsed than observed for (*E*)-**1** (ACUVAV, Mata & Peris, 2001 ▸; IBAXAM, DeHope *et al.*, 2011 ▸; IVOSER, Skibar *et al.*, 2004 ▸; JANJAJ, Dong *et al.*, 1989 ▸; OJUXEY, Roemer *et al.*, 2016 ▸; QICKIW, Chen *et al.*, 2000 ▸; REBDAD; WIMYOH, Nagahora *et al.*, 2007 ▸, Roemer & Lentz, 2008 ▸, Farrugia *et al.*, 2009 ▸). The 1,1-diferrocenylethene structure also has eclipsed Cp rings. The two staggered (*E*)-configured examples have a bulky substituent on one of the distal Cp rings; those with a less bulky Cp substituent have the eclipsed arrangement. Inter­estingly, (*E*)-**1** has no Cp substit­uents yet the Cp ring arrangement deviates significantly from eclipsed. The degree of eclipsing of the Cp conformations found among the mol­ecules with the (*Z*)-configuration, two of which have a cyclo­propene ring as the ethene bridge (AMODIP, Klimova, Berestneva, Ramirez *et al.*, 2003 ▸; EQOMIG, Klimova, Berestneva, Cinqu­antini *et al.*, 2003 ▸), is more varied (AMODOV and AMODUB, Klimova, Berestneva, Ramirez *et al.*, 2003 ▸; BADDAM, Beletskaya *et al.*, 2001 ▸, Solntsev *et al.*, 2011 ▸; JAJYIF and JAJYOL, García, Flores-Alamo, Flores & Klimova, 2017 ▸; KIGQUO, Klimova *et al.*, 2013 ▸; LUFCEW, García *et al.*, 2014 ▸; QASPEI, QATDAT and QATDEX, García, Flores-Alamo, Ortiz-Frade & Klimova, 2017 ▸; QICKOC, Chen *et al.*, 2000 ▸; TUJDEI, Klimova *et al.*, 2009 ▸).

## Synthesis and crystallization   

The title compound was prepared according to the reaction sequence presented in the scheme below[Chem scheme2]. A solution of thio­ketone **2** (297 mg, 1 mmol; prepared according to Mlostoń *et al.*, 2015 ▸) in THF (3 ml) was cooled to 198 K (acetone/dry ice). Then, TMS-CHN_2_ was added portion-wise to the mixture until the green colour of the starting thio­ketone disappeared. The magnetically stirred reaction mixture was allowed to warm slowly to *ca* 268 to 273 K and at this temperature a commercially available solution of tetra­butyl­ammonium fluoride (TBAF, 1 ml, 1 *M*) was added in small portions. Stirring was continued for 20 min, and after warming to room temperature, the solvent was evaporated under vacuum. The crude product was analyzed by ^1^H NMR spectroscopy, which revealed the presence of two isomeric ethenes in a ratio of *ca* 10:1. After column chromatography (SiO_2_, CH_2_Cl_2_/hexane 3:7), the major product was isolated, contaminated with a small admixture of the minor one, as an analytically pure sample (78% yield). After additional crystallization from a hexa­ne/CH_2_Cl_2_ mixture, 285 mg (54%) of pure (*E*)-**1** were isolated as orange crystals with m.p. 485–487 K. From this material, crystals suitable for the X-ray diffraction measurements were separated without additional recrystallization.
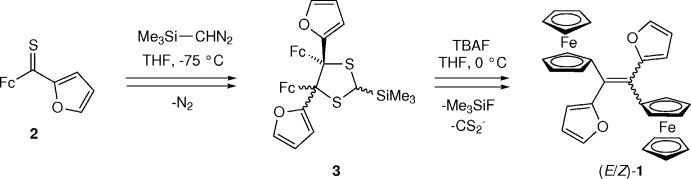


^1^H NMR [600 MHz, CDCl_3_, *δ* (ppm), *J* (Hz)]: 3.63–3.65 [*m*, 4CH(Fc)], 4.13–4.15 [*m*, 4CH(Fc)], 4.16 [*s*, 10CH(Fc)], 6.40 [*d*, ^3^ *J*_H,H_ = 3.0, 2CH(Fur)], 6.54 [*dd*, ^4^ *J*_H,H_ = 1.8, ^3^ *J*_H,H_ = 3.0, 2CH(Fur)], 7.58 [*brs*, 2CH(Fur)]. ^13^C NMR [150 MHz, CDCl_3_, *δ* (ppm)]: 68.5, 68.9 [2 signals for 8CH(Fc)], 69.6 [10CH(Fc)], 85.4 [2C(Fc)], 109.1, 111.1, 140.8 [3 signals for 6CH(Fur)], 129.1 (C=C), 153.3 [2C(Fur)]. ESI–MS (mixture of isomers): 528 (100, [*M*]^+^), 529 (50, [*M* + 1]^+^). Elemental analysis calculated for C_30_H_24_Fe_2_O_2_ (528.20): C 68.22, H 4.58%; found: C 68.38, H 4.61%.

## Refinement   

Crystal data, data collection and structure refinement details are summarized in Table 2 ▸. All H atoms were placed in geometrically calculated positions and were constrained to ride on their parent atom with C—H = 0.95 Å and with *U*_iso_(H) = 1.2*U*_eq_(C).

## Supplementary Material

Crystal structure: contains datablock(s) I, global. DOI: 10.1107/S2056989018005078/sj5552sup1.cif

Structure factors: contains datablock(s) I. DOI: 10.1107/S2056989018005078/sj5552Isup2.hkl

Supporting information file. DOI: 10.1107/S2056989018005078/sj5552Isup3.cdx

Supporting information file. DOI: 10.1107/S2056989018005078/sj5552Isup4.cdx

CCDC reference: 1833400

Additional supporting information:  crystallographic information; 3D view; checkCIF report

## Figures and Tables

**Table 1:** Hydrogen-bond geometry (Å, °) *Cg*1, *Cg*2 and *Cg*3 are the centroids of the C6–C10, C11–C15 and C2/O1/C3–C5 rings, respectively.

*D*—H⋯*A*	*D*—H	H⋯*A*	*D*⋯*A*	*D*—H⋯*A*
C3—H3⋯*Cg*1^i^	0.95	2.81	3.686 (3)	153
C8—H8⋯*Cg*2^ii^	0.95	2.85	3.764 (2)	161
C10—H10⋯*Cg*3^iii^	0.95	2.68	3.2097 (18)	116

Symmetry codes: (i) 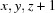; (ii) 
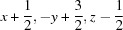
; (iii) 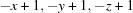.

**Table 2:** Experimental details

Crystal data	
Chemical formula	[Fe_2_(C_5_H_5_)_2_(C_20_H_14_O_2_)]
*M*_r_	528.19
Crystal system, space group	Monoclinic, *P*2_1_/*n*
Temperature (K)	160
*a*, *b*, *c* (Å)	5.81006 (13), 22.7138 (5), 8.38031 (18)
β (°)	91.785 (2)
*V* (Å^3^)	1105.40 (4)
*Z*	2
Radiation type	Mo *K*α
μ (mm^−1^)	1.34
Crystal size (mm)	0.20 × 0.16 × 0.08

Data collection	
Diffractometer	Oxford Diffraction SuperNova, dual-radiation diffractometer
Absorption correction	Multi-scan (*CrysAlis PRO*; Rigaku OD, 2015 ▸)
*T*_min_, *T*_max_	0.895, 1.000
No. of measured, independent and observed [*I* > 2σ(*I*)] reflections	13881, 3021, 2612
*R*_int_	0.026
(sin θ/λ)_max_ (Å^−1^)	0.708

Refinement	
*R*[*F* ^2^ > 2σ(*F* ^2^)], *wR*(*F* ^2^), *S*	0.032, 0.077, 1.04
No. of reflections	3021
No. of parameters	154
H-atom treatment	H-atom parameters constrained
Δρ_max_, Δρ_min_ (e Å^−3^)	0.49, −0.36

Computer programs: *CrysAlis PRO* (Rigaku OD, 2015 ▸), *SHELXT2014* (Sheldrick, 2015*a* ▸), *SHELXL2014* (Sheldrick, 2015*b* ▸), *ORTEPII* (Johnson, 1976 ▸), *Mercury* (Macrae *et al.*, 2006 ▸), *PLATON* (Spek, 2015 ▸) and *publCIF* (Westrip, 2010 ▸).

## supplementary crystallographic information

### Crystal data

**Table:**

[Fe_2_(C_5_H_5_)_2_(C_20_H_14_O_2_)]	*F*(000) = 544
*M**_r_* = 528.19	*D*_x_ = 1.587 Mg m^−^^3^
Monoclinic, *P*2_1_/*n*	Mo *K*α radiation, λ = 0.71073 Å
*a* = 5.81006 (13) Å	Cell parameters from 7365 reflections
*b* = 22.7138 (5) Å	θ = 3.0–29.9°
*c* = 8.38031 (18) Å	µ = 1.34 mm^−^^1^
β = 91.785 (2)°	*T* = 160 K
*V* = 1105.40 (4) Å^3^	Tablet, orange
*Z* = 2	0.20 × 0.16 × 0.08 mm

### Data collection

**Table:**

Oxford Diffraction SuperNova, dual radiation diffractometer	3021 independent reflections
Radiation source: SuperNova (Mo) X-ray Source	2612 reflections with *I* > 2σ(*I*)
Mirror monochromator	*R*_int_ = 0.026
Detector resolution: 10.3801 pixels mm^-1^	θ_max_ = 30.2°, θ_min_ = 2.6°
ω scans	*h* = −8→7
Absorption correction: multi-scan (CrysAlisPro; Rigaku OD, 2015)	*k* = −31→31
*T*_min_ = 0.895, *T*_max_ = 1.000	*l* = −11→11
13881 measured reflections	

### Refinement

**Table:**

Refinement on *F*^2^	0 restraints
Least-squares matrix: full	Hydrogen site location: inferred from neighbouring sites
*R*[*F*^2^ > 2σ(*F*^2^)] = 0.032	H-atom parameters constrained
*wR*(*F*^2^) = 0.077	*w* = 1/[σ^2^(*F*_o_^2^) + (0.0291*P*)^2^ + 0.9916*P*] where *P* = (*F*_o_^2^ + 2*F*_c_^2^)/3
*S* = 1.04	(Δ/σ)_max_ = 0.001
3021 reflections	Δρ_max_ = 0.49 e Å^−^^3^
154 parameters	Δρ_min_ = −0.36 e Å^−^^3^

### Special details

**Table:**

Experimental. Data collection and full structure determination done by Prof. Anthony Linden: anthony.linden@chem.uzh.chSolvent used: hexane / dichloromethane Cooling Device: Oxford Instruments Cryojet XL Crystal mount: on a glass fibre Frames collected: 1290 Seconds exposure per frame: 10.0 Degrees rotation per frame: 1602.0 Crystal-detector distance (mm): 55.0
Geometry. All esds (except the esd in the dihedral angle between two l.s. planes) are estimated using the full covariance matrix. The cell esds are taken into account individually in the estimation of esds in distances, angles and torsion angles; correlations between esds in cell parameters are only used when they are defined by crystal symmetry. An approximate (isotropic) treatment of cell esds is used for estimating esds involving l.s. planes.

### Fractional atomic coordinates and isotropic or equivalent isotropic displacement parameters (Å^2^)

**Table:**

	*x*	*y*	*z*	*U*_iso_*/*U*_eq_	
Fe1	0.46176 (4)	0.65950 (2)	0.41357 (3)	0.01767 (8)	
O1	0.5980 (2)	0.54191 (6)	0.79034 (16)	0.0295 (3)	
C1	0.5597 (3)	0.52572 (7)	0.5053 (2)	0.0159 (3)	
C2	0.7063 (3)	0.53842 (7)	0.6485 (2)	0.0187 (3)	
C3	0.7668 (5)	0.55207 (10)	0.9050 (3)	0.0408 (6)	
H3	0.7408	0.5562	1.0158	0.049*	
C4	0.9717 (4)	0.55529 (10)	0.8395 (3)	0.0418 (6)	
H4	1.1150	0.5624	0.8938	0.050*	
C5	0.9354 (3)	0.54589 (8)	0.6699 (2)	0.0256 (4)	
H5	1.0487	0.5451	0.5906	0.031*	
C6	0.5501 (3)	0.57204 (7)	0.38168 (19)	0.0168 (3)	
C7	0.7351 (3)	0.61094 (7)	0.3435 (2)	0.0212 (3)	
H7	0.8841	0.6111	0.3937	0.025*	
C8	0.6590 (4)	0.64909 (8)	0.2187 (2)	0.0264 (4)	
H8	0.7483	0.6788	0.1701	0.032*	
C9	0.4265 (4)	0.63519 (8)	0.1791 (2)	0.0262 (4)	
H9	0.3324	0.6541	0.0998	0.031*	
C10	0.3583 (3)	0.58793 (7)	0.2787 (2)	0.0200 (3)	
H10	0.2106	0.5700	0.2772	0.024*	
C11	0.4528 (4)	0.68024 (9)	0.6520 (2)	0.0295 (4)	
H11	0.5075	0.6563	0.7382	0.035*	
C12	0.5838 (3)	0.72260 (9)	0.5685 (2)	0.0310 (4)	
H12	0.7412	0.7320	0.5891	0.037*	
C13	0.4377 (4)	0.74812 (8)	0.4493 (2)	0.0286 (4)	
H13	0.4798	0.7778	0.3758	0.034*	
C14	0.2176 (3)	0.72175 (8)	0.4587 (2)	0.0267 (4)	
H14	0.0866	0.7305	0.3922	0.032*	
C15	0.2268 (3)	0.68009 (8)	0.5840 (2)	0.0268 (4)	
H15	0.1028	0.6562	0.6169	0.032*	

### Atomic displacement parameters (Å^2^)

**Table:**

	*U*^11^	*U*^22^	*U*^33^	*U*^12^	*U*^13^	*U*^23^
Fe1	0.02204 (13)	0.01261 (12)	0.01843 (13)	0.00067 (9)	0.00192 (9)	−0.00138 (9)
O1	0.0359 (8)	0.0287 (7)	0.0239 (7)	0.0074 (6)	0.0035 (6)	−0.0018 (6)
C1	0.0146 (7)	0.0145 (7)	0.0186 (8)	0.0026 (6)	0.0019 (6)	−0.0016 (6)
C2	0.0225 (8)	0.0125 (7)	0.0211 (8)	0.0001 (6)	0.0004 (6)	−0.0008 (6)
C3	0.0646 (16)	0.0329 (11)	0.0242 (10)	0.0080 (11)	−0.0104 (10)	−0.0070 (9)
C4	0.0466 (13)	0.0289 (11)	0.0480 (13)	−0.0068 (10)	−0.0295 (11)	0.0027 (10)
C5	0.0207 (8)	0.0236 (9)	0.0324 (10)	−0.0016 (7)	−0.0022 (7)	0.0050 (8)
C6	0.0192 (8)	0.0130 (7)	0.0184 (8)	0.0008 (6)	0.0028 (6)	−0.0025 (6)
C7	0.0214 (8)	0.0167 (8)	0.0260 (9)	0.0003 (6)	0.0064 (7)	−0.0014 (7)
C8	0.0377 (10)	0.0189 (8)	0.0233 (9)	−0.0002 (7)	0.0127 (8)	0.0012 (7)
C9	0.0414 (11)	0.0192 (8)	0.0179 (8)	0.0057 (8)	−0.0011 (7)	−0.0012 (7)
C10	0.0242 (8)	0.0153 (8)	0.0204 (8)	0.0020 (6)	−0.0022 (7)	−0.0037 (6)
C11	0.0464 (12)	0.0227 (9)	0.0193 (8)	0.0082 (8)	−0.0015 (8)	−0.0057 (7)
C12	0.0278 (10)	0.0274 (10)	0.0378 (11)	−0.0027 (8)	0.0019 (8)	−0.0172 (8)
C13	0.0407 (11)	0.0133 (8)	0.0324 (10)	0.0001 (7)	0.0121 (8)	−0.0032 (7)
C14	0.0282 (9)	0.0233 (9)	0.0285 (9)	0.0079 (7)	0.0006 (8)	−0.0050 (7)
C15	0.0326 (10)	0.0219 (9)	0.0267 (9)	−0.0035 (7)	0.0116 (8)	−0.0063 (7)

### Geometric parameters (Å, º)

**Table:**

Fe1—C7	2.0352 (17)	C5—H5	0.9500
Fe1—C8	2.0380 (18)	C6—C10	1.434 (2)
Fe1—C13	2.0404 (18)	C6—C7	1.435 (2)
Fe1—C9	2.0456 (18)	C7—C8	1.419 (3)
Fe1—C12	2.0457 (19)	C7—H7	0.9500
Fe1—C14	2.0465 (18)	C8—C9	1.417 (3)
Fe1—C11	2.0555 (19)	C8—H8	0.9500
Fe1—C10	2.0591 (17)	C9—C10	1.424 (3)
Fe1—C15	2.0605 (18)	C9—H9	0.9500
Fe1—C6	2.0712 (16)	C10—H10	0.9500
O1—C2	1.365 (2)	C11—C15	1.415 (3)
O1—C3	1.371 (3)	C11—C12	1.424 (3)
C1—C1^i^	1.360 (3)	C11—H11	0.9500
C1—C6	1.476 (2)	C12—C13	1.415 (3)
C1—C2	1.478 (2)	C12—H12	0.9500
C2—C5	1.349 (2)	C13—C14	1.416 (3)
C3—C4	1.329 (4)	C13—H13	0.9500
C3—H3	0.9500	C14—C15	1.414 (3)
C4—C5	1.446 (3)	C14—H14	0.9500
C4—H4	0.9500	C15—H15	0.9500
			
C7—Fe1—C8	40.77 (7)	C10—C6—C7	106.52 (15)
C7—Fe1—C13	129.40 (8)	C10—C6—C1	127.83 (15)
C8—Fe1—C13	105.96 (8)	C7—C6—C1	125.64 (15)
C7—Fe1—C9	68.43 (8)	C10—C6—Fe1	69.23 (9)
C8—Fe1—C9	40.59 (8)	C7—C6—Fe1	68.20 (9)
C13—Fe1—C9	113.70 (8)	C1—C6—Fe1	126.65 (11)
C7—Fe1—C12	107.73 (8)	C8—C7—C6	108.76 (16)
C8—Fe1—C12	113.42 (8)	C8—C7—Fe1	69.73 (10)
C13—Fe1—C12	40.51 (8)	C6—C7—Fe1	70.89 (9)
C9—Fe1—C12	145.21 (8)	C8—C7—H7	125.6
C7—Fe1—C14	168.36 (8)	C6—C7—H7	125.6
C8—Fe1—C14	129.68 (8)	Fe1—C7—H7	125.3
C13—Fe1—C14	40.56 (8)	C9—C8—C7	108.05 (16)
C9—Fe1—C14	108.34 (8)	C9—C8—Fe1	69.99 (11)
C12—Fe1—C14	68.12 (8)	C7—C8—Fe1	69.51 (10)
C7—Fe1—C11	116.63 (8)	C9—C8—H8	126.0
C8—Fe1—C11	146.90 (9)	C7—C8—H8	126.0
C13—Fe1—C11	68.12 (8)	Fe1—C8—H8	126.1
C9—Fe1—C11	172.39 (9)	C8—C9—C10	108.15 (16)
C12—Fe1—C11	40.64 (8)	C8—C9—Fe1	69.42 (10)
C14—Fe1—C11	67.88 (8)	C10—C9—Fe1	70.22 (10)
C7—Fe1—C10	68.35 (7)	C8—C9—H9	125.9
C8—Fe1—C10	68.30 (7)	C10—C9—H9	125.9
C13—Fe1—C10	147.05 (8)	Fe1—C9—H9	126.0
C9—Fe1—C10	40.58 (7)	C9—C10—C6	108.50 (16)
C12—Fe1—C10	172.33 (8)	C9—C10—Fe1	69.20 (10)
C14—Fe1—C10	117.03 (7)	C6—C10—Fe1	70.13 (9)
C11—Fe1—C10	134.35 (8)	C9—C10—H10	125.8
C7—Fe1—C15	149.65 (8)	C6—C10—H10	125.8
C8—Fe1—C15	169.52 (8)	Fe1—C10—H10	126.5
C13—Fe1—C15	67.94 (8)	C15—C11—C12	107.88 (18)
C9—Fe1—C15	132.74 (8)	C15—C11—Fe1	70.08 (11)
C12—Fe1—C15	67.98 (8)	C12—C11—Fe1	69.31 (11)
C14—Fe1—C15	40.27 (8)	C15—C11—H11	126.1
C11—Fe1—C15	40.22 (8)	C12—C11—H11	126.1
C10—Fe1—C15	111.80 (8)	Fe1—C11—H11	126.1
C7—Fe1—C6	40.91 (7)	C13—C12—C11	107.82 (18)
C8—Fe1—C6	68.75 (7)	C13—C12—Fe1	69.54 (11)
C13—Fe1—C6	169.58 (8)	C11—C12—Fe1	70.05 (11)
C9—Fe1—C6	68.58 (7)	C13—C12—H12	126.1
C12—Fe1—C6	132.19 (8)	C11—C12—H12	126.1
C14—Fe1—C6	149.60 (7)	Fe1—C12—H12	125.9
C11—Fe1—C6	111.05 (7)	C12—C13—C14	108.09 (17)
C10—Fe1—C6	40.64 (6)	C12—C13—Fe1	69.94 (11)
C15—Fe1—C6	118.64 (7)	C14—C13—Fe1	69.95 (11)
C2—O1—C3	106.35 (17)	C12—C13—H13	126.0
C1^i^—C1—C6	123.93 (19)	C14—C13—H13	126.0
C1^i^—C1—C2	120.04 (19)	Fe1—C13—H13	125.7
C6—C1—C2	116.02 (14)	C15—C14—C13	108.12 (17)
C5—C2—O1	110.90 (16)	C15—C14—Fe1	70.40 (11)
C5—C2—C1	132.40 (16)	C13—C14—Fe1	69.49 (11)
O1—C2—C1	116.67 (15)	C15—C14—H14	125.9
C4—C3—O1	110.49 (19)	C13—C14—H14	125.9
C4—C3—H3	124.8	Fe1—C14—H14	125.7
O1—C3—H3	124.8	C14—C15—C11	108.10 (17)
C3—C4—C5	107.03 (18)	C14—C15—Fe1	69.33 (10)
C3—C4—H4	126.5	C11—C15—Fe1	69.70 (11)
C5—C4—H4	126.5	C14—C15—H15	126.0
C2—C5—C4	105.22 (18)	C11—C15—H15	126.0
C2—C5—H5	127.4	Fe1—C15—H15	126.6
C4—C5—H5	127.4		
			
C3—O1—C2—C5	0.1 (2)	Fe1—C8—C9—C10	−59.76 (12)
C3—O1—C2—C1	178.20 (15)	C7—C8—C9—Fe1	59.30 (12)
C1^i^—C1—C2—C5	115.8 (2)	C8—C9—C10—C6	0.0 (2)
C6—C1—C2—C5	−65.4 (2)	Fe1—C9—C10—C6	−59.30 (12)
C1^i^—C1—C2—O1	−61.8 (2)	C8—C9—C10—Fe1	59.26 (13)
C6—C1—C2—O1	117.02 (16)	C7—C6—C10—C9	0.52 (19)
C2—O1—C3—C4	0.4 (2)	C1—C6—C10—C9	179.64 (16)
O1—C3—C4—C5	−0.7 (3)	Fe1—C6—C10—C9	58.72 (12)
O1—C2—C5—C4	−0.5 (2)	C7—C6—C10—Fe1	−58.21 (11)
C1—C2—C5—C4	−178.21 (18)	C1—C6—C10—Fe1	120.92 (17)
C3—C4—C5—C2	0.7 (2)	C15—C11—C12—C13	−0.2 (2)
C1^i^—C1—C6—C10	32.9 (3)	Fe1—C11—C12—C13	59.52 (13)
C2—C1—C6—C10	−145.89 (16)	C15—C11—C12—Fe1	−59.70 (13)
C1^i^—C1—C6—C7	−148.2 (2)	C11—C12—C13—C14	−0.1 (2)
C2—C1—C6—C7	33.1 (2)	Fe1—C12—C13—C14	59.79 (13)
C1^i^—C1—C6—Fe1	123.9 (2)	C11—C12—C13—Fe1	−59.84 (13)
C2—C1—C6—Fe1	−54.85 (19)	C12—C13—C14—C15	0.3 (2)
C10—C6—C7—C8	−0.81 (19)	Fe1—C13—C14—C15	60.05 (13)
C1—C6—C7—C8	−179.96 (15)	C12—C13—C14—Fe1	−59.78 (13)
Fe1—C6—C7—C8	−59.67 (12)	C13—C14—C15—C11	−0.4 (2)
C10—C6—C7—Fe1	58.86 (11)	Fe1—C14—C15—C11	59.11 (13)
C1—C6—C7—Fe1	−120.29 (16)	C13—C14—C15—Fe1	−59.48 (13)
C6—C7—C8—C9	0.8 (2)	C12—C11—C15—C14	0.3 (2)
Fe1—C7—C8—C9	−59.60 (13)	Fe1—C11—C15—C14	−58.88 (13)
C6—C7—C8—Fe1	60.40 (12)	C12—C11—C15—Fe1	59.22 (13)
C7—C8—C9—C10	−0.5 (2)		

Symmetry code: (i) −*x*+1, −*y*+1, −*z*+1.

### Hydrogen-bond geometry (Å, º)

Cg1, Cg2 and Cg3 are the centroids of the C6–C10, C11–C15 and C2/O1/C3–C5 rings, respectively.

**Table:**

*D*—H···*A*	*D*—H	H···*A*	*D*···*A*	*D*—H···*A*
C3—H3···*Cg*1^ii^	0.95	2.81	3.686 (3)	153
C8—H8···*Cg*2^iii^	0.95	2.85	3.764 (2)	161
C10—H10···*Cg*3^i^	0.95	2.68	3.2097 (18)	116

Symmetry codes: (i) −*x*+1, −*y*+1, −*z*+1; (ii) *x*, *y*, *z*+1; (iii) *x*+1/2, −*y*+3/2, *z*−1/2.
